# Addition of Molasses Ameliorates Water and Bio-Floc Quality in Shrimp Pond Water

**DOI:** 10.21315/tlsr2022.33.1.8

**Published:** 2022-03-31

**Authors:** Yustian Rovi Alfiansah, Jens Harder, Matthew James Slater, Astrid Gärdes

**Affiliations:** 1 The Alfred Wegener Institute (AWI), Helmholtz Centre for Polar and Marine Research, Am Handelshafen 12, Bremerhaven, 27570, Germany; 2 Research Center for Oceanography, the National Research and Innovation Agency (BRIN), Jalan Pasir Putih I, Ancol Timur, Jakarta Utara, 14430 Indonesia; 3 Leibniz Centre for Tropical Marine Research (ZMT), Fahrenheitstraße 6, Bremen, 28359, Germany; 4 Department of Molecular Ecology, Max Planck Institute for Marine Microbiology (MPI), Celsiusstraße 1, Bremen, 28359 Germany

**Keywords:** *Penaeus vannamei*, Particulate Organic Matter, Bacterial Colonisation, Bacterial Lifestyle

## Abstract

Suspended particulate matter, phytoplankton and bacteria can be exploited to form larger aggregates, so-called bio-flocs. These serve as feeds for cultured shrimps, govern inorganic nutrients and load bacteria including pathogens. The current study aimed to simulate aggregate formation from available particulate matter in shrimp pond water and investigate quality of aggregates as well as possible impact to the pond water. Molasses was added to cylindrical tanks containing shrimp pond waters, and the tanks were rolled for 48 h. Besides water quality (inorganic nutrients and physical parameters), the researchers investigated and separated bacterial community compositions (BCC) to free-living (FL) and bio-flocs/particle-attached (PA) bacteria via 16S rRNA amplicon sequencing, and measured macro-molecules contents (carbohydrates, lipids and proteins) in the bio-flocs. Molasses addition increased bacterial numbers in the bio-flocs to two-fold higher than the FL’s. Moreover, potential probiotics such as *Halomonas*, *Psychrobacter*, *Mesonia* and *Chromohalobacter* were detected associated to bio-flocs and dominated the BCC. In contrast, bio-flocs without molasses showed 4-fold less carbohydrates and harboured elevated potential pathogens such as *Vibrio* and *Alteromonas*. Results show that molasses (at C/N ratio 1:2) increases pH (to 8.2 ± 0.09 and 8.0 ± 0.04 after 24 h and 48 h, respectively) in pond water, improving beneficial biofloc formation. Molasses also increased carbohydrates and proteins in bio-flocs and maintained abundances of beneficial bacteria resulting in low inorganic nutrient concentrations. Thus, molasses is suitable for shrimp farming to improve rearing processes.

**Highlight**

- Molasses increased pH and C/N ratio resulting in more nutritious bio-flocs and potential beneficial bacteria in shrimp pond waters.
- The potential beneficial bacteria such as Exiguobacterium, Halomonas Psychrobacter and Salegentibacter increased, while opportunistic pathogenic ones such as Vibrio and Alteromonas decreased.
- Molasses, as one of soluble carbon sources can be applied in shrimp farming for both pond and recirculating aquaculture systems to form bio-flocs from available suspended particles in water.

## INTRODUCTION

Regular feed input in shrimp ponds results not only in shrimp growth, but also in rise of suspended particulate matter and inorganic nutrients which may initiate blooms of heterotrophic bacteria and phytoplankton/microalgae (Funge-Smith & Briggs 1998; De Schryver *et al*. 2008). Microalgae may assimilate inorganic nutrients and provide oxygen through photosynthesis (Shaari *et al*. 2011; Yaakob *et al*. 2014; Kumar & Babu 2015). Bacteria including heterotrophic bacteria such as *Halomonas*, *Nitrosomonas*, *Nitrobacter*, *Pseudomonas* and *Sulfitobacter* share identical roles as of microalgae (Moriarty 1997), for example involve in nitrification, organic matter decomposition and sulphur oxidation (Ray *et al*. 2010; Sombatjinda *et al*. 2011; Sangnoi *et al*. 2016; Xiao *et al*. 2017). They have been reported as an additional or even a substitutive feed in shrimp aquaculture (Ray *et al*. 2010; Hargreaves 2013; Suantika *et al*. 2013). Thus, above elements can be generated to form organic aggregates, so-called bio-flocs. Then, they can be used to nourish cultured biota such as shrimps or fish.

An approach to improve aquaculture, so-called bio-floc technology, has been developed based on the aggregation of suspended particles including organic matter and microorganism such as bacteria, fungi, microalgae, zooplankton and protozoa (De Schryver *et al*. 2008; Hargreaves 2013). In shrimp aquaculture, the presence of suspended particles at size between 0.5 μm to 5 μm increased shrimp growth rates up to 53% over growth rates attained in clear well water, while particles greater than 5.0 μm improved growth by an additional 36% (Moss 2002). Aggregates of suspended particles or bio-flocs can be naturally generated in pond aquaculture due to water movement and high concentrations of organic matter, bacteria and microalgae (Hargreaves 2013; Avnimelech 2015). This approach has been preliminary applied in Tilapia farming with successfully yield of cultured biota (Avnimelech 2015).

Several factors affect aggregation of particulate matter, and microbial attachment and settlement. Water movement, presence of particular exudates such as exopolysaccharides in the water column will affect aggregate sizes (Simon *et al*. 2002), while random motion of microorganisms and biological process like bacterial competition for nutrient and space affect bacterial diversity and abundance (Kiørboe 2001; Kiørboe *et al*. 2003; Grossart *et al*. 2004). In addition, aggregate formation may also occur by particular interaction between microalgae (i.e., diatom) with heterotrophic bacteria, for example *Marinobacter* spp induces *Thalassiosira weissflogii* aggregation (Gärdes *et al*. 2011). Furthermore, the aggregates may attract bacteria which live freely in surrounding water column, so-called free-living (FL) bacteria, to attach onto aggregates (Kiørboe *et al*. 2003; Kramer *et al*. 2013). Thus, the FL bacteria then turn to be particle-attached (or aggregate-associated) bacteria (Grossart 2010).

Beyond the beneficial microorganisms present in shrimp pond waters, opportunistic pathogenic bacteria, which may cause shrimp diseases (Hasson *et al*. 2009; Joshi *et al*. 2014) have been reported to be present in the pond water too (Zhang, Wang, *et al*. 2014; Xiong *et al*. 2015; Heenatigala & Fernando 2016; Zheng *et al*. 2016; Alfiansah *et al*. 2020). Among pathogens, *Vibrio* has been commonly reported as the causative agent of shrimp diseases (Mastan 2015). Furthermore, *V. parahaemolyticus* provokes lethal diseases in shrimp (Munro *et al*. 2003; Soto-Rodriguez *et al*. 2015). Since shrimp ponds contain elevated densities of microalgae and bacteria (Moriarty 1997; Alonso-Rodrıguez & Paez-Osuna 2003), these microorganisms may become target to improve shrimp cultivation.

Considering the presence of heterotrophic bacteria including beneficial and opportunistic bacteria, high abundance of microalgae and organic matter, and potential continuous bio-floc formation in shrimp farming, it is necessary to investigate bio-floc formation as well as quality of bio-flocs generated from shrimp pond water. Avnimelech (2015) reported that addition of carbohydrates such as tapioca and wheat or rice bran accelerated bio-floc formation as well as growth of heterotrophic bacteria. Furthermore, addition of molasses has been reported to improve bio-floc quality in fresh-water prawn *Macrobrachium rosenbergii* (Miao *et al*. 2017). Since the addition of rice-bran or flour-related carbohydrates may increase suspended particulate matter which in turn may give potential drawback for pond aquacultures, it then is necessary to investigate the application of easy dissolved carbohydrates such as molasses in *P. vannamei* pond farming. In addition, thorough investigation of bacterial community compositions is needed to understand dominant bacteria in pond water because if bio-flocs load pathogenic bacteria, it may menace shrimp aquacultures.

This study aims to generate bio-flocs after addition of molasses in shrimp pond waters and therein changes in bacterial communities towards beneficial bacteria as well as to assess the impact of molasses addition in pond waters. We attempted to examine the indigenous bacterial community dynamics in the bio-flocs (PA) and FL form as well as pond water quality including bioflocs. We hypothesise that the molasses increases bacterial numbers, affects bacterial community compositions (BCC) in pond waters and improves the pond water quality including the bio-flocs.

## MATERIALS AND METHODS

### Experimental Design

**Figure 1:**
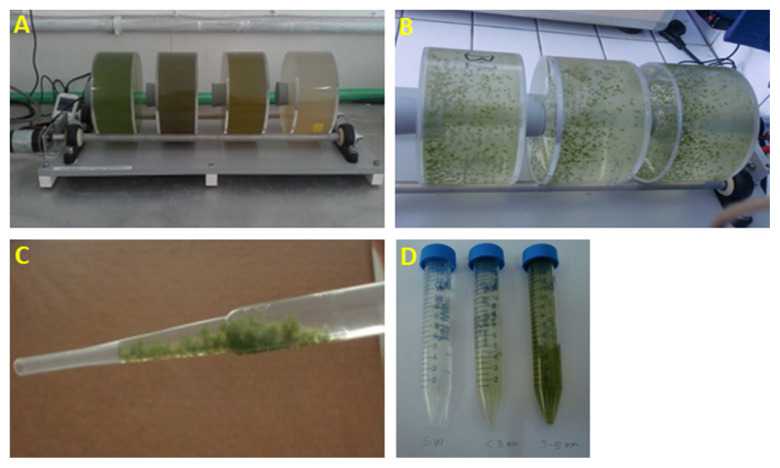
Rolling tank experiments to generate aggregate formation. (A) Rolling tanks containing shrimp pond water with molasses (brown) and without molasses (green); (B) aggregates without molasses in cylindrical tanks; (C) collection of aggregates; (D) collected aggregates based on their sizes which are smaller than 3 mm and in size of 3 mm to 5 mm.

Rolling-tank experiments were performed in Diponegoro University, Semarang, Indonesia on November 2016. Twelve litres of pond water at 60th day of shrimp rearing were collected from a shrimp pond in Rembang Regency, Central Java, Indonesia (−6°42′11.66″S 111°21′54″E) on November 2016 and placed into 12 cylindrical tanks, 1 L per each tank (Fig. 1A). Six tanks served as control and the others were treated with the addition of 1 mL (equivalent to 1.4 g dry weight mL^−1^) molasses per tank. All tanks were rolled in roller tables (Gärdes *et al*. 2011) to generate bio-flocs at 12 rpm per min for 48 h in room temperature (29°C) and darkness. Samplings were conducted at 24 h and 48 h by sacrificing 3 tanks per treatment. Water samples at day of culturing 60th days was chosen because they started to contain high density of bacteria and phytoplankton (as indicated by chlorophyll a concentration), in amount of 7.41 ± 0.36 log10 CFU mL^−1^ and 33.7 ± 14.2 mg mL^−1^, respectively (Alfiansah *et al*. 2018).

### Water Quality Measurements

Physical water parameters such as salinity, temperature, and pH were measured *ex-situ*, using calibrated Manta Eureka 2 multi-probes (Eureka Environmental Engineering, Texas, USA). Measurements of inorganic nutrients (ammonium, nitrite, nitrate, phosphate, and silicate) were performed at the Research Center for Oceanography, BRIN, Jakarta Indonesia, while suspended particulate matter (SPM), total carbon/nitrogen (C/N), C-organic carbon (C-org) content, and transparent exopolymer particles (TEP) were analysed at the Center for Tropical Marine Research (ZMT) Bremen, Germany.

Measurement procedures were as follow, the inorganic nutrient measurements were done in triplicates according to the colorimetry method by Strickland and Parsons (1972), with a Shimadzu UV-1800 spectrophotometer. SPM was measured as dry mass on pre-combusted GF/F filters (porosity 0.7 μm, ∅︀ 47 mm, VWR, France) in triplicates after filtration of 500 mL water sample. Weight of the filters (SPM) was determined using a precision balance (ME 36S, Sartorius, Göttingen, Germany) after drying the filters for 24 h at 40°C. Half of the filter were cut and used for total carbon (TC) and total nitrogen (TN) measurement, while another half was for particulate organic carbon (C-org) measurement. The C-org was measured after acidification of the filter with 1N HCl to remove the inorganic carbon. CN ratios were calculated from C-org and TN according to USDA (2011). Total carbon (TC), total nitrogen (TN), and C-org concentrations were measured on an Elemental Analyzer (EA-3000, EuroVector, Italy).

TEP analysis was performed for 100 mL macro aggregate-free water filtered onto 0.4 μm pore size polycarbonate filters (Whatman, Dassel, Germany), using a spectrophotometric method introduced by Alldredge *et al*. (1998), with an updated protocol by Engel (2009). Briefly, this method relates the adsorption of an Alcian blue dye to the weight of polysaccharides filtered on 0.4 μm pore size polycarbonate filters (Whatman, Dassel, Germany). A calibration curve was prepared using the reference polysaccharide gum xanthan from *Xanthomonas campestris* cultures.

### Bio-floc Collection

Clumps of bio-flocs in each tank (Fig. 1B) were collected using disposable pipettes (Fig. 1C), placed in 15 mL centrifuge tubes, and settled for 15 min (Fig. 1D). Water in upper layer of collected bio-flocs was discarded. Total bioflocs were weighted using an analytical balance (ME 36S, Sartorius, Göttingen, Germany). From these bio-flocs, 100 μL were used for bacterial counting for bio-floc (PA) fraction using a DAPI method as mentioned in following section, while 500 μL were used for bacterial community analysis on particle-attached (bio-flocs). Both samples were filtered on 3 μm, ∅︀ 47 mm polycarbonate filters. Remaining bio-flocs and filters were stored in −20°C until macromolecule content (such as C/N, carbohydrate, lipid and protein content) and bacterial analyses (genomic DNA extraction and cell counting), respectively, while water in each tank (macro aggregate-free water) was used for water quality and bacterial community analyses for the FL fraction which were explained below.

### Organic Macromolecule (C/N total, carbohydrates, lipid and protein) Contents in Bio-flocs

Samples for the organic macromolecule analyses were prepared from harvested bio-flocs. Two millilitres (2 mL) of bio-flocs were centrifuged at 1000xg for 5 min to form a pellet. Wet weight of the pellets was measured using a precision balance (ME 36S, Sartorius, Göttingen, Germany). Due to insufficient amount of pelleted material for C/N total measurement, samples from replicates were pooled and then analysed as mentioned before. Carbohydrate and lipid measurements were performed according to (De Coen & Janssen 1997), while protein analyses were done according to Bradford (1976). Samples were prepared as follow: pellets of bio-flocs were homogenated in phosphate buffer (pH 7.4) in dilution factor 1:5 to 1:6. Homogenates were then sonicated for 2 min using a sonicator (Sonophis Bendelin HD 3100, Germany). From these, 150 μL of homogenate was collected for carbohydrates and protein measurements, while 150 μL–300 μL of homogenate was collected for lipids.

Samples for carbohydrates and proteins were precipitated using 50 μL of 15%-trichloro-acetic acid (TCA), incubated in −20°C for 10 min and centrifuged at 1000× *g* at 4°C for 10 min. Supernatant was collected for carbohydrate measurement, while the pellet was dissolved in 1 M NaOH for protein measurement. Serial dilution of D-glucose and bovine serum albumin (BSA) served as standard curve for carbohydrates and proteins, respectively (see Appendix A for the supplementary information). For carbohydrates, 50 μL of the 5% phenol and 200 μL of concentrated sulphuric acid (98% H_2_SO_4_) were added into 50 μL supernatant, followed by incubation at room temperature (27°C) for 30 min, while for proteins 270 μL Bradford reagent was added into 30 μL of adjusted sample (pH 6–8) and then incubated as previous condition. Optical density of the samples was measured using a spectrophotometer (Infinite M200 Pro, Tecan, Germany) at 492 nm and 592 nm for carbohydrates and proteins, respectively.

Lipid fraction from 150 μL homogenates was extracted with 250 μL chloroform and 250 μL methanol and centrifuged at 1000× *g* for 5 min. The extracts were transferred into glass tubes, followed by addition of 500 μL 98% H_2_SO_4_. Samples were incubated in an oven at 200°C for 20 min, and added with 1.5 mL distillate water after samples reached room temperature. The total of 300 μL samples were measured spectrophotometrically using the spectrophotometer at 375 nm.

### Bacterial Cell Measurement

Bacterial cell numbers were counted using a 4′,6-diamidino-2-phenylindole (DAPI) staining method according to Kepner and Pratt (1994). Briefly, filters containing bacterial cells were stained with 1 μg mL^−1^ of DAPI solution for 5 min, then washed in 80% ethanol and rinsed with sterile distillate water. Stained filters were air dried in the dark for 30 min, and then mounted with 10 μL of mounting solution consisting of 3:1 Citifluor AF mounting medium (Citifluor Ltd, London, UK) and Vecta shield (Vector Laboratories Inc., Burlingame, USA). Bacterial cells observation was performed with a fluorescence microscope *Axio Imager. D2* (Zeiss, Jena, Germany) at 1000× magnification. Bacterial cell abundance was calculated from 30 photos per filter, using the free software *ImageJ (*https://imagej.nih.gov/ij/index.html, Ducret *et al*. 2016).

### Samples for Bacterial Community Analyses

Bacterial community compositions from the PA fraction were collected from bio-flocs as mentioned above. FL bacteria fraction was collected by filtering filtrate of TEP samples on 0.2 μm polycarbonate filter with a diameter 47 mm and 25 mm in amount 100 mL and 200 μL for bacterial community analysis and cell counting, respectively. The filters were then stored in −20°C until analyses.

### Molecular Analysis of Bacterial Communities

Genomic DNAs from bio-floc (PA) and FL fraction of water samples were extracted using the phenol-chloroform-isoamyl alcohol method, according to Nercessian *et al*. (2005). DNA pellets were dissolved in 40 μl TE buffer (10 mM Tris-HCl, 1 mM EDTA, pH 8.5). DNA concentrations were measured photometrically and checked for purity (ratio of light absorption at 260 to 280 nm) using a spectrophotometer plate reader (Infinite M200 Pro, Tecan, Germany). Genomic DNAs in concentration between 0.2–100 ng μL^−1^ were then sent for 16S rRNA gene amplification and sequencing at LGC genomics (Berlin, Germany).

16S rRNA gene amplification was performed from genomic DNA extracts. DNA sequences of the V3–V4 hypervariable region of the 16S rRNA gene were obtained from amplicon sequencing with the primer set S-D-Bact-0314-b-S-17 (5′-CCTACGGGNGGCWGCAG-3′)/S-D-Bact-0785-a-A-21 (5′-GACTACHVGGGTATCTAAKCC-3′) (Klindworth *et al*. 2013). Sequencing at LGC genomics (Berlin) was done on an Illumina MiSeq using the V3 Chemistry (Illumina) in a 2 × 300 bp paired-end run. Demultiplexing, i.e., grouping of sequences by sample, and the removal of the primer sequences from the raw paired-end reads were performed by LGC genomics (Berlin, Germany). Sequences were quality trimmed with a sliding window of four bases and a minimum average quality of 15 with *trimmomatic* v. 033 (Bolger *et al*. 2014). Quality trimmed sequences were merged using PEAR v. 0.9.8 (Zhang, Kobert, *et al*. 2014). Minimum entropy decomposition (MED) was used to cluster sequences (Eren *et al*. 2013; Ramette & Buttigieg 2014). MED applies the principle of oligotyping (Eren *et al*. 2013), of which Shannon entropy was used to iteratively partition amplicons at single nucleotide resolution, which provide more accurate descriptions of closely related but distinct taxa (Utter *et al*. 2016). During MED, we used a minimum substantive abundance (−M) of 50 read numbers to filter low-abundant OTU with the decomposition of one nucleotide position at a time (−d 1). For each OTU, one representative sequence (node) was taxonomically classified with SINA (SILVA Incremental Aligner) v1.2.11 using the SILVA rRNA project reference database (SILVA v.128) at a minimum alignment similarity and quality of 0.9 and a last common ancestor consensus of 0.7 (Pruesse *et al*. 2012). Unwanted lineages (such as archaea, chloroplasts, and mitochondria) were removed.

### Statistical Analyses

Water quality, including physical parameters, inorganic nutrients, and bacterial cell numbers as well as organic nutrient content (carbohydrates, lipid, and protein) in bio-flocs was tested with one-way ANOVA, followed by Tukey-HSD post-hoc test. Bray-Curtis dissimilarity values were calculated to evaluate dissimilarity in the BCC. All statistical analyses were performed in R (R version 3.4.2, R Core Team, 2017, using R Studio v.0.98.1056) with the packages vegan (Oksanen *et al*. 2017).

## RESULTS

### Water Quality Parameters

At 60 days of culturing, shrimp pond waters contained important amounts of total suspended solid, phytoplankton, transparent exopolymer particles as of reported elsewhere (Alfiansah *et al*. 2018). In tanks with molasses addition, suspended particulate matter (SPM) decreased from 35.7 ± 1.9 mg L^−1^ to 4.5 ± 1.8 mg L^−1^, while SPM in control tanks decreased to 16.1 ± 3.9 mg L^−1^. Transparent exopolymer particles (TEP) declined drastically in both treatments after 24 h from 7.7 ± 0.3 mg L^−1^ to 1.4 ± 0.3 mg L^−1^ and 0.8 ± 0.2 mg L^−1^ for molasses and control tanks, respectively. pH in molasses treatment increased up to 8.1, which was moderately higher than the initial pH (7.9), while pH in control tanks decreased to 7.5. After 48 h, salinity slightly decreased to 32.64 and 33.07 in molasses and control tanks, respectively. Moreover, C-total and N-total in pond water with molasses increased up to 10-fold higher after 24 h (Table 1). Inorganic nutrient concentrations, except phosphate and silicate decreased drastically in molasses tanks after 48 h. Concentration of toxic inorganic nutrients such as N-ammonium and N-nitrite decreased below 0.1 mg L^−1^ in molasses treatment. Salinity, SPM, C-org and ammonium were among measured quality parameters which differed significantly between tank with molasses and control (one-way ANOVA, Table 1).

Total bio-flocs with molasses varied in range 6.47 ± 0.50 to 6.57 ± 0.31 mL per 1 L pond water, which were equivalent to 77.17 ± 18.30 to 93.82 ± 25.39 mg of wet weight of the bio-flocs, while in control tank, total bioflocs were 6.40 ± 0.79 to 6.93 ± 0.32 mL, which was equivalent to 86.26 ± 24.46 to 119.74 ± 26.37 mg of wet weight of the bio-flocs per 1 L shrimp pond water. The amounts of bio-flocs with molasses did not differ from those of without molasses.

Molasses contained total carbon and total nitrogen of 34.71 ± 0.60 and 0.21 ± 0.04% per mg dry weight, respectively. They contained proteins and lipids in quantity of 9.22 ± 1.56 and 81.68 ± 5.43 mJ mg^−1^, correspondingly. Once the molasses were added into pond water, concentrations of lipid, total C and total N in bio-flocs with molasses increased to 16.0% and 2.9% per mg of bioflocs per 1 L pond water after 48 h, respectively, which were slightly higher than those of in bio-flocs without molasses (Table 2). In addition, molasses increased carbohydrate content in the bio-flocs up to 4-fold higher (44.0 ± 5.4 mJ carbohydrates mg^−1^ bio-flocs). In contrast, protein and lipid contents in the bio-flocs with molasses did not differ from those of in the bio-flocs without molasses (Table 2).

The water movement forming bio-flocs and the addition of molasses influenced bacterial numbers as well as bacterial community compositions. The addition of molasses increased the bacterial cell numbers in the PA fraction, as well as in the FL fraction. After 24 h, bacterial concentrations in the PA and the FL were higher in pond water with molasses, in amount of 2.5 × 10^7^ ± 1.0 ×10^6^ and 5.5 × 10^6^ ± 2.0 × 10^5^ cell mL^−1^, respectively. However, the cell numbers decreased significantly to 4.5 × 10^6^ ± 1.0 × 10^5^ cells mL^−1^ in the FL fraction, while the cells numbers in the PA fraction stayed steady thereafter (Table 3).

### Bacterial Communities in Pond Water

**Figure 2:**
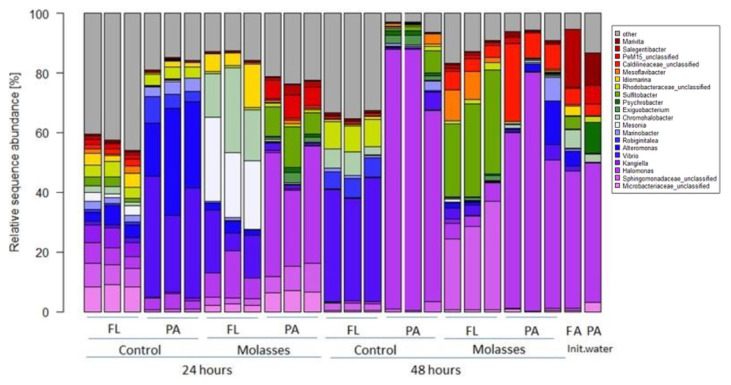
Contribution of the most abundant bacterial operational taxonomic units (OTUs) in free-living (FL) and particle-attached/bio-floc (PA) fractions in pond water without molasses (control) and with molasses within 48 h. Taxonomic affiliation for OTUs is provided for genus level. The bacterial community composition in FA of initial water (Init.water) was bacteria which were available in free-living and particle in range > 0.2 to < 3 μm, while in the FL of the control and the molasses was bacteria which were available in range 0.2 to 0.4 μm. The PA fraction included bacteria which attached to particles larger than > 3μm.

Addition of molasses changed BCC in shrimp pond waters. Dominant BC in PA (>3 μm particles) and FL to smaller particles (0.2 – < 3 μm) fraction of initial pond water were *Halomonas* and *Salegentibacter* (Fig. 1). After 24 h incubation, the BCC in the FL and the PA fraction differed from the initial BCC. *Alteromonas* and *Vibrio* became dominant bacteria in the PA fraction (bioflocs) of pond water without molasses addition. In contrast, the FL fraction shared relatively similar bacterial OTUs proportions without any dominant OTUs genera at the same sampling time. In water with molasses addition, *Halomonas* and *Sulfitobacter* were dominant bacteria in the PA fraction, while *Chromohalobacter*, *Mesonia*, *Halomonas*, and *Vibrio* dominated the FL fraction at 24 h. After 48 h, bacterial shifts occurred in the PA and FL fraction in both water samples. In water without molasses, *Halomonas* became the dominant bacteria in the PA fraction, while *Alteromonas*, *Vibrio* and OTUs of unclassified *Rhodobacteriaceae* dominated the FL fraction. In water with molasses, *Halomonas* and *Salegentibacter* remained to be the dominant bacteria in the PA fraction, while *Mesoflavibacter*, *Sulfitobacter* and OTUs of unclassified *Sphingomonadaceae* dominated the FL fraction (Fig. 2).

Bacterial community compositions (BCC) in FL and PA fraction for both shrimp pond water samples differed over the time with the dissimilarity values higher than 0.6. Moreover, the BCC of the same fraction and treatment were dissimilar between the BCC at 24 h and 48 h with the Bray-Curtis dissimilarity values higher than 0.8 (Table 4).

## DISCUSSION

Our study indicates that addition of molasses improves shrimp pond water quality in terms of pH, heterotrophic bacterial numbers, inorganic nutrient concentrations and quality of bio-flocs. These data support possible application of molasses in *P. vannamei* farming as reported to be successfully applied in *Penaeus monodon* (Panjaitan 2010) and *Macrobrachium rosenbergii* (Miao *et al*. 2017). The heterotrophic bacteria might uptake carbon source from molasses and inorganic nutrients from pond water decreasing concentrations of ammonium, nitrite and nitrate to favourable levels for shrimps, as also reported previously (Panjaitan 2010). Moreover, molasses increases C-org in pond water resulting in prominent presence of carbon source, which can be uptaken for bacterial growth. In this study, the bio-flocs formation reduced transparent exopolymer particles (TEP) and suspended particulate matter (SPM) concentrations in pond water.

Even though molasses did not increase total quantity of bio-flocs, it augmented carbohydrate contents in bio-flocs significantly. In shrimp aquaculture, molasses has been used to increase C/N ratio to form nutritive bio-flocs (Avnimelech 2015; Miao *et al*. 2017). Addition of 1 mL of molasses per L shrimp pond water (0.001 % v/v, C/N ratio = 1:2) seemed to exceed the recommended C:N ratio for bio-floc formation. Thus, the concentration of molasses as the C source need to be reduced up to 10-fold dilution. Interestingly, C:N ratio turned to 1:1 after 48 h of water rolling with slightly higher C-org and N content than those of at 24 h. We suppose that increased bacterial cells contribute to the augmentation of C-org and N content in the bio-flocs. Nutritious bio-flocs may improve shrimp diets, especially for the need of carbohydrates. The presence of high carbohydrate level (30%) associated with crude protein (35% CP) tend to improve energy retention up to 19% in shrimps (Cuzon *et al*. 2000). Moreover, rhe nutritious bio-flocs can be formed by adjusting C:N ratio at 1:15 till 1:20 (Avnimelech 2015; Miao *et al*. 2017).

Aggregates provide a suitable habitat for microorganisms (Grossart & Simon 1998; Kiørboe 2001; Simon *et al*. 2002). Our experiment indicated that the highest bacterial abundances were reached when bacteria attached to bio-flocs (PA fraction), which were 10-fold and 5-fold higher than bacterial abundances in FL fraction after 24 h and 48 h, respectively. Bacterial abundances in bio-floc fraction may decrease due to detachment, predation, bacteriabacteria inhibition or mortality due to phage (Grossart *et al*. 2004; Riemann & Grossart 2008; Kramer *et al*. 2013). Our results is in opposite to those of Kramer *et al*. (2013) and Lyons *et al*. (2010) who mentioned that bacterial numbers on aggregates remain constant over the time. We suppose that dissolved oxygen (DO) depletion due to microbial processes, such as respiration and sulphur oxidation as well as the presence of hydrogen sulphide (H_2_S), influence bacterial abundances in both fractions. We observed H_2_S in shrimp pond water due to their characteristic odour. Unfortunately, we could not measure H_2_S as well as DO concentration directly in our treatments. United States Occupational Safety and Health Administration/US OSHA (2021) indicated that H_2_S gas is in range of 0.01–1.5 ppm when rotten egg odour is released, and if the offensive rotten egg odour occurred, the maximum concentration of H_2_S is 5 ppm.

Our results correspond to previous studies (Kiørboe *et al*. 2003; Lyons *et al*. 2005), which reported that aggregates content higher bacterial abundance and diversity, including pathogenic bacteria than those of in adjacent water column. Bacteria may use aggregates as source of nutrient and shelter from destructing physical factors such as pH and salinity (Lyons *et al*. 2010; Kramer *et al*. 2013), however the presence of toxic substance, such as H_2_S or depletion of oxygen in surrounding water may limit heterotrophic bacterial growth. In this study, the addition of molasses ameliorated the growth of beneficial bacteria. The beneficial bacteria, such as *Halomonas*, *Salegentibacter*, *Psychrobacter*, *Mesonia* and *Chromohalobacter* dominated BCC in both fractions of molasses containing shrimp pond water. In comparison, potential pathogenic bacteria such as *Alteromonas* and *Vibrio* dominated the BCC of pond water without molasses addition. The beneficial bacteria may serve several roles for shrimp aquaculture. For instance, *Halomonas* is involved in the nitrification process and serves as a probiotic for cultured shrimps (Zhang *et al*. 2009; Suantika *et al*. 2013; Sangnoi *et al*. 2016), while *Salegentibacter* and *Psychrobacter* participate in organic matter degradation (Li *et al*. 2017) and may have potential biotechnological applications for example as an enzyme producer (Kimbrel *et al*. 2018).

Bio-flocs in shrimp pond waters may attract bacteria to attach resulting in concentrated bacterial cells in the bio-flocs (PA fraction). FL bacteria may utilise dissolved organic matter (DOM), for example transparent exopolymer particles (TEP) for cell multiplication. The FL bacteria are usually motile and have chemotactic behaviour which facilitate efficient attachment or detachment to substrates/particles or organisms (Grossart 2010). Once the FL bacteria attach to particles, they change their lifestyle to particle-attached bacteria. At this stage, they may escape from several disrupting factors such as UV light, predation or limited nutrients. The PA bacteria may proliferate and dominate bacterial communities over the time being, as shown by *Halomonas* in the bio-flocs with molasses. However, their dominance may depend on the environmental parameters as well as the presence of other bacterial taxa.

Change of dominant bacteria in the bio-flocs may occur if environmental parameters change or other bacteria dominate the competition for substrate/habitat or benefit from environmental changes, as also reported previously (Kramer *et al. 2013*; Ortega-Retuerta *et al*. 2013; Rieck *et al*. 2015; Alfiansah *et al*. 2018). Moreover, addition of beneficial bacterial strains such as *Bacillus*, *Nitrococcus*, *Nitrospira* and *Lactococcus* into bio-flocs may also increase probiotic contents (Zhang *et al*. 2009; Suantika *et al*. 2013). Thus, bio-flocs having beneficial bacteria and sufficient contents of macromolecules are promising natural food for shrimp farming. They can be applied in various farming stages covering shrimp hatchery as well as grow-out ponds.

## CONCLUSIONS

The formation of bio-flocs from suspended particulate matter, transparent exopolymer particles and microorganisms increases natural food supply for shrimps and facilitates particle removal from water columns through sedimentation or sinking of larger particles. Bio-floc quality can be improved by addition of external carbon such as molasses. It also allows buffering of water pH, increases carbon content in pond water, and supports bacterial and phytoplankton growth, which ultimately governs inorganic nutrient concentrations in shrimp ponds. To obtain rich nutritious bio-flocs containing dominant beneficial indigenous bacteria, the C:N ratio in pond water and the presence of pathogenic bacteria has to be determined and measured carefully. Further feeding experiments using different sizes of bio-flocs would be promising to understand the impact of natural feed source (bio-flocs) on shrimp performance as well as to identify bacterial load and diversity in the respective size of bio-flocs.

## Figures and Tables

**Table 1:** Physical parameters, carbon and nitrogen contents and inorganic nutrient concentrations in water from rolling tank experiment.

Parameters	Samples					Sig.

	Initial pond water	Control		Molasses		
	
		24 h	48 h	24 h	48 h	
Temperature (°C)	30.24 ± 0.09	29.43 ± 0.09	29.32 ± 0.02	29.36 ± 0.03	29.31 ± 0.02	
Salinity (psu)	33.64 ± 0.17^a^	33.31 ± 0.05^ab^	33.07 ± 0.18^b^	32.75 ± 0.21^c^	32.64 ± 0.07^c^	***
pH	7.90 ± 0.02^a^	7.50 ± 0.09^ab^	7.20 ± 0.05^b^	8.20 ± 0.09^c^	8.00 ± 0.04^a^	***
TEP (mg L^−1^)	7.70 ± 0.40^a^	0.80 ± 0.20^b^	1.80 ± 0.30^c^	1.40 ± 0.30^c^	3.40 ± 1.70^d^	***
SPM (mg L^−1^)	35.70 ± 1.90^a^	18.00 ± 2.40^b^	16.10 ± 3.90^b^	5.10 ± 1.90^c^	4.50 ± 1.80^c^	***
C-org (% dry weight SPM)	2.40 ± 0.20^a^	5.00 ± 0.70^a^	3.10 ± 0.60^a^	7.50 ± 2.20^b^	7.60 ± 0.50^b^	***
C total (% dry weight SPM)	2.57 ± 0.58	19.38 ± 25.09	2.74 ± 0.56	25.45 ± 9.91	30.18 ± 14.58	
N total (% dry weight SPM)	0.38 ± 0.07	3.29 ± 4.41	0.47 ± 0.12	4.48 ± 2.08	5.27 ± 2.80	
C:N ratio	1:6	NA	1:6	1:2	1:1	
*Inorganic nutrients (mg/L)*						
Ammonium	0.63 ± 0.09^a^	0.39 ± 0.13^b^	0.17 ± 0.06^c^	0.07 ± 0.04^d^	0.007 ± 0.002^e^	***
Nitrate	0.68 ± 0.27^a^	0.03 ± 0.02^b^	0.001 ± 0.0008^c^	0.01 ± 0.009^b^	0.004 ± 0.003^d^	***
Nitrite	0.03 ± 0.003^a^	0.12 ± 0.04^b^	0.002 ± 0.001^c^	0.01 ± 0.007^a^	0.002 ± 0.0002^c^	***
Phosphate	1.46 ± 0.89	0.55 ± 0.15	0.56 ± 0.04	0.63 ± 0.03	0.52 ± 0.03	***
Silicate	0.76 ± 0.33^a^	0.25 ± 0.02^b^	0.27 ± 0.03^b^	0.53 ± 0.09^a^	0.37 ± 0.03^b^	**

*Notes*: Differences in ANOVA were indicated with asterix; superscript letters indicate significant differences after pairwise *t*-test. Data are shown as mean ± standard deviation. Sig. = significance *p*-value in ANOVA; ^***^ = < 0.001; ^**^ = < 0.01; NA = Not applied due to high standard deviation of samples.

**Table 2:** Volume and macromolecule contents (carbohydrates, lipids and proteins) in molasses and bio-flocs.

Parameters	Molasses	Bio-flocs				*p*-value

		Control		With molasses		
	
		24 h	48 h	24 h	48 h	
Volume (mL L^−1^)	1	6.4 ± 0.8	6.9 ± 0.3	6.6 ± 0.3	6.5 ± 0.5	
Wet weight (g)	1.2 ± 0.2	86.3 ± 24.5*	119.7 ± 26.4*	77.2 ± 18.3*	93.8 ± 25.4*	< 0.01
C total (% per mg dw)	34.7 ± 0.6	10.1	13.7	12.0	16.0	NA
N total (% per mg dw)	0.21 ± 0.04	1.7	2.2	1.9	2.9	NA
Carbohydrates (mJ mg^−1^)	> 100	13.6 ± 2.7^a^	9.7 ± 1.8^a^	9.4 ± 8.3^a^	44.0 ± 5.4^b^	< 0.01
Lipid (mJ mg^−1^)	81.7 ± 5.4	58.9 ± 2.8	69.0 ± 7.5	89.3 ± 36.5	56.1 ± 9.5	0.22
Protein (mJ mg^−1^)	9.2 ± 1.6	30.0 ± 5.4	50.8 ± 5.9	49.2 ± 27.3	71.7 ± 11.9	0.06

*Notes*:

* = bio-flocs after centrifugation at 1000*x*g for 5 min, dw = dry weight, C and N content is from a pool of triplicates.

Superscripts after values indicate a significant difference.

**Table 3:** Concentrations of bacterial cells in FL and bio-flocs from rolling tank experiments with and without molasses. Different superscripts in a row indicate differences at *t*-test.

Fraction	24 h		48 h	
	
	Control	Molasses	Control	Molasses
Bio-flocs/particle-attached (in 10^7^ cell/mL)	1.6 ± 0.2^a^	2.5 ± 0.1^b^	2.2 ± 0.1^b^	2.3 ± 0.2^b^
FL (in 10^6^ cell/mL)	3.9 ± 0.2^a^	5.5 ± 0.2^b^	3.3 ± 0.1^c^	4.5 ± 0.1^d^

**Table 4:** Bray-Curtis dissimilarity values of bacterial community composition in free-living (FL) and particle-attached (PA) fraction of water without molasses (control/C) and with molasses (M) at 24 h (1d) and 48 h (2d).

Samples	C_FL-1d	C_PA-1d	C_FL-2d	Samples	M_FL-1d	M_PA-1d	M_FL-2d
C_FL-1d				M_FL-1d			
C_PA-1d	0.80			M_PA-1d	0.88		
C_FL-2d	0.79	0.68		M_FL-2d	0.89	0.63	
C_PA-2d	0.86	0.90	0.94	M_PA-2d	0.87	0.86	0.86

## APPENDIX A

### Supplementary Information 1: Dilution of standard for carbohydrates, lipids and proteins, and wavelength for spectrophotometer

**Table:**

Standards	Concentration (mg/mL)	Wavelengths (nm)
Carbohydrates (D-Glucose)	0.25	492
	0.125	
	0.0625	
	0.032	
	0.016	
	0.0078	
	0	
Proteins (Bovine Serum Albumin)	0.5	592
	0.25	
	0.125	
	0.0625	
	0.0313	
	0.0155	
	0	
Lipids (Glyceryl Tripalmitate)	2.4	375
	1.6	
	0.8	
	0.4	
	0.2	
	0.1	
	0.05	
	0	
